# An electronic medical records-based approach to identify idiosyncratic drug-induced liver injury in children

**DOI:** 10.1038/s41598-019-54075-4

**Published:** 2019-12-02

**Authors:** Tracy L. Sandritter, Jennifer L. Goldman, Clayton J. Habiger, James F. Daniel, Jennifer Lowry, Ryan T. Fischer

**Affiliations:** 1 Children’s Mercy Hospital, Division of Clinical Pharmacology, Toxicology, and Therapeutic Innovation, Kansas City, MO USA; 2 Children’s Mercy Hospital, Division of Infectious Disease, Kansas City, MO USA; 3 The University of Tennessee Health Science Center, Department of Pediatrics, Memphis, TN USA; 4 Children’s Mercy Hospital, Division of Gastroenterology, Kansas City, MO USA

**Keywords:** Autoimmune hepatitis, Hepatotoxicity

## Abstract

Drug-induced liver injury (DILI) is the leading cause of liver failure in the United States and the most common cause of drug recall. As opposed to the recognized direct toxicity of super-therapeutic acetaminophen or chemotherapeutic agents in children, limited data exists for pediatric populations on the incidence of idiosyncratic DILI (iDILI) that may develop independently of drug dose or duration of administration. To improve the detection of adverse drug reactions at our hospital, we utilized electronic medical records-based automated trigger tools to alert providers of potential iDILI. Clinical criteria concerning for iDILI were defined as serum ALT > 5x or serum bilirubin > 1.5x upper limit of normal in the setting of medication exposure. Over a two year period, 12 patients were identified as having possible or probable iDILI. Out of the identified patients, three were males, and the mean age was 10.8 years. Implicated agents included eight antibiotics, two anti-epileptics, one anti-psychotic, and one anti-inflammatory medication. Roussel-Uclaf Causality Assessment Methods identified one “possible” case, 11 “probable” cases, and one “highly probable” case of iDILI. Improved awareness and more vigilant programming can generate better data on iDILI and improve our understanding of the condition and its incidence in children.

## Introduction

Drug-induced liver injury (DILI) is the leading cause of acute liver failure in the United States^1^ and the most common cause of drug recall from the market^2^. Recent data demonstrate that in adult patients with confirmed DILI, one in 10 will need to undergo transplantation and one in six will have evidence of long term liver injury six months after initial suspected damage^3^. In an analysis by Mindikoglu and colleagues of 73,977 patients (adults and children) that underwent transplant from October of 1987 to December of 2006, they identified 661 cases of drug-induced acute liver failure. Ninety-four of these were in children, with acetaminophen being responsible for the largest portion of cases (29%)^4^. The Pediatric Acute Liver Failure Study Group showed that DILI accounted for 16% of acute liver failure cases in the United States^5^. Again, the most common etiology was acetaminophen overdose (12% of all acute liver failure), and 4% were due to non-acetaminophen drug injury^5^.

While the direct hepatotoxicity of super-therapeutic acetaminophen is well-described^6^, the less frequent, idiosyncratic causes of DILI (iDILI) in pediatric patients remain poorly understood. Exposure to antimicrobials, antiepileptics, antidepressants, and medications for attention deficit disorders represent most iDILI cases in children^4,7,8^. Active surveillance of pediatric iDILI is rare. The diagnosis and—as importantly—the determination of iDILI causality remains a significant obstacle in clinical practice. This difficulty can be attributed to several key factors. Clinically, biochemically, and histologically, iDILI can resemble many forms of both acute and chronic liver injury. Definitive laboratory studies are lacking, further complicating the diagnosis, and determination of drug causality is limited to scoring systems that were not designed for pediatric patients.

Children’s Mercy Hospital (CMH) is a 354-bed, tertiary care, free-standing children’s hospital in Kansas City, MO. To better characterize and detect possible adverse drug reactions (ADR) in children, our hospital developed a campus-wide pharmacovigilance service, the Drug Safety Service (DSS) in October 2010. The DSS is operated by a dedicated clinical pharmacist in the Division of Clinical Pharmacology, Toxicology, and Therapeutic Innovation with the support of the Pharmacy Department. A detailed description of the development and operation of the program has been previously described^9^. The goal of the program is to identify and improve documentation of potential adverse drug reaction cases, and in turn, better understand medications associated with toxicity and possible preventive strategies. To further enhance the identification of ADR, specifically related to DILI, EMR triggers were developed and implemented in 2012 to screen patients for potential DILI. Herein, we assess two years of cases associated with pediatric iDILI and report on the implicated agents as well as associated clinical and laboratory characteristics in our population.

## Results

**Figure 1:**
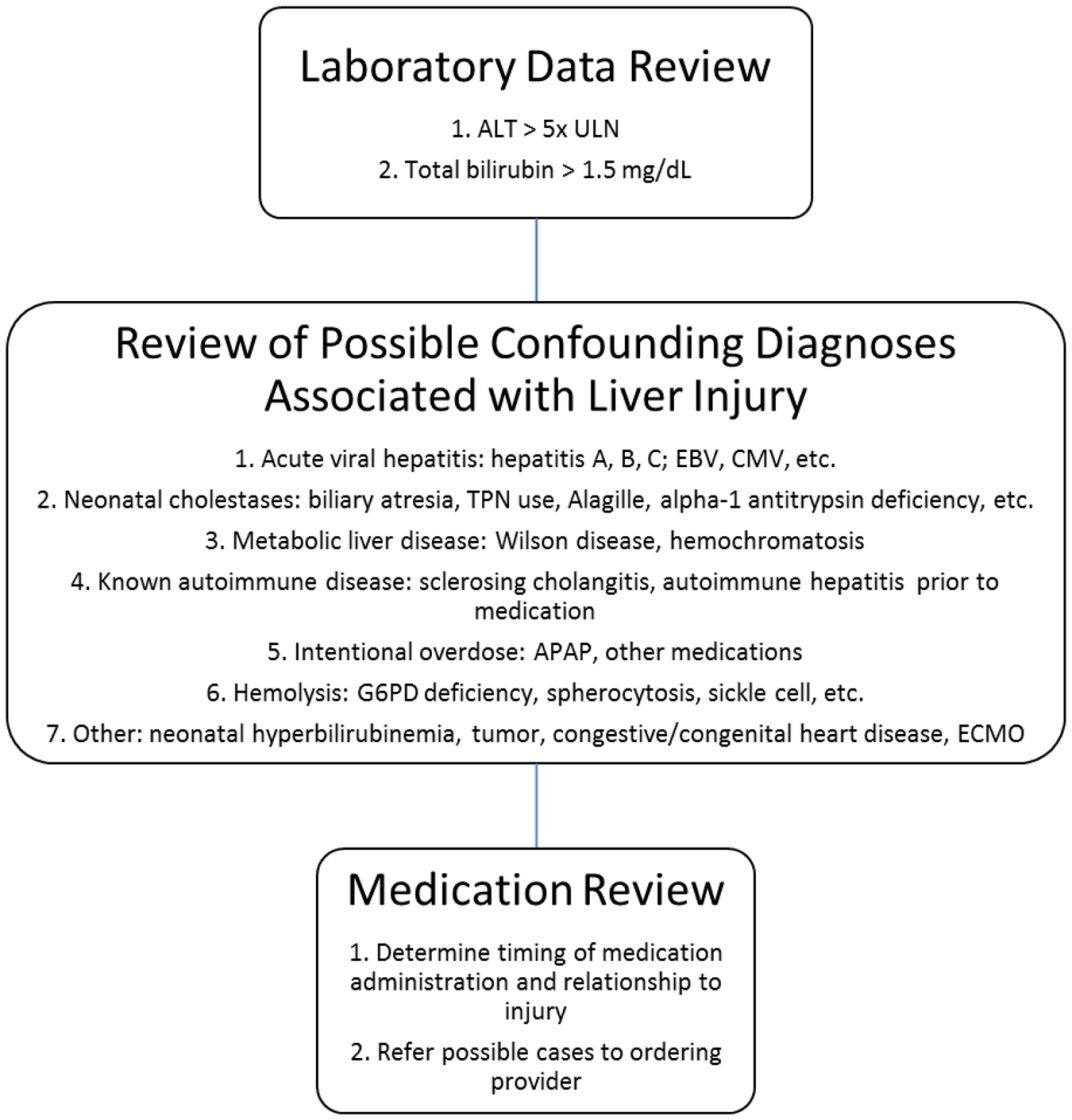
Workflow for the identification of possible idiosyncratic drug-induced liver injury.

A total of 3,275 inpatient and outpatient lab values were assessed during the study period with 791 and 2,484 values triggering alanine aminotransferase (ALT) and bilirubin criteria, respectively (Fig. 1). Following the exclusion of redundant patients (triggers of the same type in the same patient that corresponded to an already assessed case) a total of 1,515 unique events remained with 453 ALT and 1,062 bilirubin triggers. Both values were triggered simultaneously in 90 patients. This represents a mean of 3.0 unique triggers to review per week day averaging five minutes per review. Of the unique ALT triggers, 2.6% (n = 12) were considered possible or probable iDILI (Table 1). However, 26% of these ALT triggers were associated with chemotherapy. When chemotherapy ALT triggers were excluded, possible or probable iDILI rose to 3.4%. Of the non-chemotherapy bilirubin triggers, only 0.5% (n = 5) were associated with iDILI. No cases of iDILI were associated with an isolated bilirubin trigger as all patients had an elevated ALT.

**Table 1:** Percent of ALT elevations associated with underlying causes.

Probable Etiologies of ALT Triggers		
	Number	Percent of Total
All Events	453	*100.0*
Chemotherapy	118	*26.0*
Infection	68	*15.0*
Trauma	29	*6.4*
Congenital Liver Disease	23	*5.1*
Cardiac Disease	22	*4.8*
Musculoskeletal Disease	15	*3.3*
Sepsis	15	*3.3*
Cholelithiasis/Cholecystitis	14	*3.1*
**DILI**	**12**	***2.6***
Parenteral Nutrition Use	12	*2.6*
Unknown	40	*8.8*
Miscellaneous*	85	*18.7*

*Includes diagnoses with fewer than 10 cases (e.g., cystic fibrosis, liver transplant, etc.).

Overall, 67,817 ALT values and 75,306 bilirubin levels were obtained across the inpatient and outpatient settings in our hospital system during the examined years. Focusing on ALT values, these were obtained from 27,130 unique patients in the two years studied. Thus, only 1.6% of the unique patients with ALT levels drawn had a triggering ALT (n = 453), and only 0.04% (n = 12) had iDILI. Interestingly, the inpatient setting accounted for only 8% (n = 2,182) of unique patient ALT levels, but 55% (n = 253) of the observed ALT triggers.

### iDILI clinical characteristics

Twelve possible or probable cases of iDILI were found in our assessment and their characteristics are listed in Table 2. Majority of patients were female (N = 9; 75%) and in early adolescence with an average age of 10.8 years. Symptoms at the time of presentation varied significantly; fever (n = 7), fatigue (n = 6), rash, abdominal pain, jaundice (n = 5 for each), arthralgia, nausea/vomiting, and pruritus (n = 4 for each) were experienced by patients (Table 3). The majority of patients displayed a hepatocellular injury pattern based on the application of the Roussel-Uclaf Causality Assessment Method (RUCAM)^10,11^. The RUCAM assessment first defines whether an injury is “hepatocellular,” “mixed,” or “cholestatic” based on the “R ratio.” Nine patients had hepatocellular injury with a minority presenting with a mixed pattern (n = 3). No patients presented with a cholestatic injury pattern alone, as defined by the R ratio (Table 2). Six patients were evaluated for iDILI in the inpatient setting, with five of those patient admissions being related to iDILI. Four hospitalized patients had iDILI with hypersensitivity features, including two with drug reaction with eosinophilia and systemic symptoms (DRESS) and one that developed hemophagocytic lymphohistiocytosis (HLH). The four patients with hypersensitivity reactions required steroids and hospitalization, while the HLH patient was the only one to develop an associated coagulopathy. No patient developed liver failure in our series, nor did any patient undergo liver biopsy.

**Table 2:** Demographic and clinical features of iDILI patients.

iDILI Patient Characteristics			
	Hospitalized (n = 6)	Outpatient (n = 6)	p-value
Female gender	5 (83%)	4 (67%)	
Mean age (years)	10.2	11.4	*0.74*
Injury type (based on R ratio*)			
Hepatitis	5	4	
Cholestatic	0	0	
Mixed	1	2	
Mean peak ALT (U/L)	1251.7	898.5	*0.6*
Mean peak bilirubin (gm/dL)	4.6	2.1	*0.23*
Mean eosinophils (cells/mcL)	0.36	0.12	*0.27*
Etiology			
*Antimicrobials*	***3***	***5***	
Oxacillin	1		
Minocycline		2	
Doxycycline		1	
Azithromycin	1		
Trimethroprim		1	
Trimethoprim- sulfamethoxazole	1		
Amoxicillin		1	
*Antiepileptics*	***2***		
Lamotrigine	1		
Carbamazepine	1		
*Antipsychotics*		***1***	
Aripiprazole		1	
*Anti-inflammatory*	***1***		
Sulfasalazine	1		
Steroid treatment	4 (67%)	0 (0%)	
Mean RUCAM	7.5	7.8	*0.65*

*R = (ALT value/ALT ULN)/(ALKP value/ALKP ULN).

R > 5 = hepatocellular, R < 2 = cholestatic, R between 2 and 5 = mixed.

**Table 3:** Reported signs and symptoms in children with iDILI.

Symptoms associated with iDILI								
Patient No.	Jaundice	Joint pain	Rash	Fever	Naseua/vomiting	Abdominal pain	Fatigue	Pruritus
1			X					X
2		X				X	X	
3	X		X	X	X	X	X	X
4		X				X	X	
5	X			X		X		
6	X	X		X			X	
7			X	X				X
8						X	X	
9		X	X	X	X			X
10								
11	X		X	X	X		X	
12	X			X	X			

### Laboratory values

Of the 12 cases of iDILI described, all 12 triggered our DSS based on ALT and five triggered based on both ALT and bilirubin levels. No cases were identified using the bilirubin trigger alone. The mean initial ALT value was 840 U/L, with the mean peak being 1075 U/L for all iDILI cases. The mean initial total bilirubin level in iDILI patients was 2.7 mg/dL with an average peak value of 3.4 mg/dL. In the majority of cases, initial ALT and bilirubin values were the peak values, and they did not vary significantly between hospitalized and non-hospitalized patients. The mean peak ALT in iDILI-related hospitalized patients was 1251.7 U/L and the mean peak ALT in non-hospitalized patients was 898.5 U/L, with a p-value of 0.6. The mean peak bilirubin in hospitalized versus non-hospitalized patients was 4.6 and 2.1, respectively (p = 0.28). These data are detailed in Table 4.

**Table 4:** Detailed clinical features of iDILI patients.

Patient	Suspected Agent	Age at iDILI (years)	Sex	Type of Injury*	Time to symptoms (days)	Time from symptoms to trigger	Peak ALT	Peak ALP	Peak GGT	Peak Bili	Eosinophils (cells x 10^3^/mcL)	RUCAM	Hospitalization (days)	Type of Reaction
1	Oxacillin	6.3	M	1	22	3	898	222	ND	0.4	0.01	7	25 (unrelated)	Hepatitis
2	Minocycline	15.3	F	1	84	0	1131	288	105	1	0	8	0	Hepatitis
3	Lamotrigine	15.9	F	3	75	33	470	294	1146	8.5	0.01	8	41	DIHS-HLH
4	Minocycline	16.8	F	1	731	15	1763	184	157	0.9	0.24	8	0	Hepatitis
5	Azithromycin	3.9	F	1	3	0	4438	546	140	10.1	0.13	7	3	Hepatitis
6	Doxycyline	16.8	F	3	92	6	344	214	136	3.4	0.03	8	0	Hepatitis
7	Sulfasalazine	3.7	F	1	21	8	463	201	ND	0.9	1.2	8	13	DIHS-DRESS
8	Aripiprazole	11.0	M	1	32	0	206	43	31	0.7	0.12	5	0	Hepatitis
9	Carbamazepine	16.9	F	1	8	3	814	69	115	0.4	0.2	7	13	DIHS
10	Trimethoprim	1.2	M	1	213	0	944	292	ND	0.3	0.12	10	0	Hepatitis
11	Trimethoprim-sulfamethoxazole	14.6	F	3	25	6	427	313	ND	7.2	0.6	8	8	DIHS-DRESS
12	Amoxicillin	7.3	F	3	8	0	1003	276	ND	6.6	0.2	8	0	Hepatitis

*(1 = hepatocellular; 2 = cholestatic; 3 = mixed).

DIHS = drug-induced hypersensitivity; HLH = hemophagocytic lymphohistiocytosis; DRESS = drug reaction with eosinophilia and systemic symptoms.

### Non-DILI ALT triggers

Further investigation revealed that ALT elevations attributed to infection (n = 68), trauma (n = 29), congenital cholestasis (biliary atresia, Alagille, etc.; n = 23), and congenital heart disease (n = 22) made up the largest proportions of patients with ALT triggers not associated with iDILI (Table 1). Investigation into the bilirubin triggers revealed that most of the non-iDILI cases were related to physiologic/human milk jaundice and hemoglobinopathies (e.g., sickle cell disease and thalassemias).

### Suspected drugs and causality assessment

Antimicrobials (n = 8) were the most prevalent drug class associated with DILI. Additionally, anti-epileptics (n = 2), anti-psychotics (n = 1) and anti-inflammatory agents (n = 1) were identified. Minocycline (n = 2) and trimethoprim-sulfamethoxazole (n = 2) were the most commonly implicated single agents (Table 4). Acne and other bacterial infections were the most common indications for suspected medication use. The average RUCAM score for all groups was 7.7.

### Temporal relationship

Following the first reported dose of a suspected medication, the median time to initial presentation of symptoms (or lab trigger if asymptomatic) was 28.5 days (range 3 to 731). The EMR trigger occurred a median of three days (range 0–33) following onset of reported symptoms. The majority of patients had a relatively benign clinical course, with no chronic liver disease identified in a median of 4.1 years of follow-up (range 1.8–5.0 years). Six patients were identified and managed in the outpatient setting and did not require hospitalization. The median number of hospital days for the six hospitalized patients was 13 (range 3–41). The median time to normalization of ALT following the recognized iDILI was 35 days, with there being no significant difference between patients managed in the inpatient setting and those seen as outpatients. Hospitalized patients normalized their ALT in an average of 19.2 days, while the ALT was noted to be in the normal range for outpatient iDILI patients after an average of 154.3 days (p = 0.15). Outpatients typically did not have the same frequency of lab draws as inpatients, and they did not receive steroids during recovery.

## Discussion

In this study, we have examined the utility of a dedicated pharmacovigilance program in detecting iDILI. In two years, 12 cases of possible, probable, or highly probable cases of liver injury were identified at our single center, a rate of detection that compares favorably to the 66 possible or probable pediatric cases identified in a 13-year, six-center study^7,8^. This is important in a number of respects. First, our study shows that iDILI detection rates may increase with implementation of an inpatient and outpatient service dedicated to identification of potential iDILI cases, and that pediatric iDILI is likely under-recognized. Two, this study emphasizes the fact that medications prescribed for both serious and relatively benign indications can lead to significant liver injury.

Detection and identification of iDILI is challenging for multiple reasons. As with other drug-induced diseases, the diagnosis of DILI is a diagnosis of exclusion or all other possible cause (e.g., Hep A, B, C infection, alpha-1-antitrypsin, Wilson’s disease). Drug exposure correlation is highly variable (days to months)^12^. The time of onset of liver dysfunction varies depending on the drug and the patient and can be long after the first ingestion of drug which might provide a false security that there is not a drug induced cause. For example minocycline and atomoxetine are both noted to have a prolonged latency (minocycline: median 569 days (range 196–647) and atomoxetine: median 510 days (range 117–699))^7^.

Additionally, unique diagnostic difficulties exist for determining iDILI in pediatrics. Specifically, the current laboratory assessment for DILI included the use of alkaline phosphate. Alkaline phosphatase is expressed in liver tissues and increases during cholestasis. However, increased serum alkaline phosphate can be a result of accelerated bone growth and excessive enzyme secretion by osteoblasts in children and therefore is a poor marker of DILI in children^13^. The RUCAM, the primary DILI causality assessment tool, requires the measurement of the alkaline phosphatase (ALKP) to ALT ratio to characterize injury. Substituting measurement of gamma-glutamyl transferase (GGT) for ALKP should be studied in iDILI causality assessments. We noted that the GGT, when measured, increased in conjunction with the transaminases and bilirubin. Some cases of cholestasis had higher GGT elevations than others. We would presume that GGT may more accurately correspond to a cholestatic iDILI response relative to alkaline phosphatase. Unfortunately, GGT is not part of the standard liver panel at many centers (including our own) and we have limited data on GGT significance in DILI. The necessary integration of an unreliable marker (alkaline phosphatase) in pediatric patients may confound the score and merits the development of a novel pediatric causality assessments.

Liver biopsy may be helpful in identifying microscopic changes that differentiate DILI from liver injury of other origins, though it is not an obligatory part of the DILI workup^14^. DILI may present histologically in the form of a number of different liver diseases, and biopsies may be non-specific. As reviewed by Teschke and Frenzel^15^, anywhere from 13–65% of patients in DILI case series from the past two decades undergo biopsy. Interestingly, no patient in our series underwent liver biopsy. A rapid recovery in most patients was observed following withdrawal of the implicated drug, sometimes in combination with immunosuppressive therapy, thus eliminating the necessity of biopsy. While biopsy may indeed be important in assessing more chronic forms of liver injury (or in differentiating severe—and potentially treatable—autoimmune disease from other forms of injury) drug withdrawal and monitoring were sufficient in our group. We recommend approaching the need for biopsy on a case-by-case basis, as a lack of histologic specificity may not justify procedural risk^15^.

Of note, chronic liver injury (defined as continued injury six months after initial diagnosis by the DILI Network^16^) did not develop in any of our patients to the best of our knowledge. One patient in the group died from complications of respiratory failure and sepsis in the setting of progressive dysautonomia associated with a previous traumatic brain injury. Hepatitis and liver dysfunction did not contribute to the death in that case. Overall, our patients would compare favorably to the 17% rate of chronic DILI reported in adults^16^, recognizing that older age is a recognized risk factor for the development of chronic DILI^17^. They would also compare favorably to Kumar and colleague’s report, where 11% of pediatric patients had a DILI-related death, and 14% went on to develop chronic disease. However, this report was notable in that three-fourths of cases were caused by complementary and alternative medicines and anti-tuberculosis agents^18^. These agents were not identified in our population. In contrast, in an updated report from the DILI Network that analyzed pediatric patients in the United States, liver failure was noted in 5% of iDILI patients, and chronic disease developed in 17% of patients^7,8^. We must acknowledge that our long-term assessment was based on up to five years of chart follow-up, and not on a repeat clinic visit, imaging or lab values after normalization of transaminases. Chronic injury can be asymptomatic, and specific screening for long-term complications (e.g., steatosis, peliosis hepatitis, nodular regenerative hyperplasia, etc.^17^) was not done.

As in other reports on pediatric DILI, antimicrobials were the most prevalent drug class associated with iDILI in our population^7,8^. Minocycline, used for the treatment of acne, was implicated twice in our identified cases of iDILI. All patients had a positive anti-nuclear antibody (ANA) screen, and symptoms suggesting an autoimmune presentation, including rash, athralgias, and fatigue. DILI attributed to minocycline is well recognized^19–21^ and continues to be a common example of drug-induced autoimmune hepatitis^7,8,22,23^. A Cochrane database study revealed that minocycline did not have a superior efficacy compared to other tetracyclines, however, and that the risk of autoimmune-type side effects of increased with duration of use^24^. Another review found doxycycline was efficacious as minocycline but with a smaller decreased risk of DILI^25^. We conclude that regular liver enzyme monitoring should be implemented with minocycline and—if possible—avoiding the medication altogether for the treatment of acne may be prudent.

This study is limited by its retrospective design and by the fact it represents cases at a single center. Also, our criteria for DILI were based on the work of Aithal and colleagues^14^, and do not necessarily reflect the same criteria set forth by other centers and consortia^7,8,26^. The lack of specific biomarkers makes the diagnosis of DILI dependent on the interpretation of laboratory, radiographic and/or histologic investigations to rule-out other causes of liver disease. Such interpretation might rely on expert consensus. For example, the DILI Network provided the following criteria for identifying iDILI in patients older than two years: clinical suspicion of hepatotoxicity combined with an aspartate aminotransferase (AST) or ALT of >5 times the ULN for the local center (or >5 times the pre-drug average) or ALKP > 2 times the ULN or pre-drug average, or serum bilirubin > 2.5 mg/dL along with any elevations in AST, ALT, or ALKP levels, or an internationalized normal ratio (INR) > 1.5 with any elevations in AST, ALT, or ALKP levels. And, ultimately, causality was determined by their own expert committee, with the DILIN Causality Committee using both RUCAM and the DILIN Causality Assessment score to determine the presence of DILI^7,8,26^.

Other published methods of identifying iDILI in large electronic record datasets have been explored. Methods have included searching for iDILI using the International Classification of Diseases-9^th^ edition or -10^th^ edition (ICD-9 or ICD-10) codes that are widely used to classify patients’ principal diagnoses. However, the codes lack specificity in the ICD-9 and ICD-10 index for iDILI, and the system relies on the medical knowledge of the coding staff to correctly enter the diagnosis^27,28^. Text searching methods may be an additional tool to identify iDILI, but efforts require specific language in patient notes, and case detection sensitivity may be sacrificed with increased search term efficiency^29^. That said, the approach our center used in identifying iDILI may be useful in future multi-center studies—perhaps in combination with previously examined strategies—to better identify and collect data on patients.

The majority of pediatric DILI reports have focused on hospitalized cases of DILI^7,8,30^. Our ADR mechanisms were able to detect potential iDILI in affiliated outpatient settings in addition to hospitalized patients. Thus, less severe, but perhaps no less important cases of iDILI were identified. In two years of iDILI identification, half of all cases (including one patient that sustained liver injury without any symptoms) were managed solely in the outpatient setting. Drug discontinuation was the only treatment necessary for these patients. Understanding what factors play roles in preventing hospitalizations related to iDILI, and quickly predicting cases that may progress to more serious disease is an imperative area of further study.

## Conclusion

DILI represents a potentially life-threatening and preventable cause of liver injury. Better mechanisms for understanding and predicting injury are desperately needed to prevent harm to thousands of children annually. We reviewed our experience with a hospital-wide adverse drug reaction reporting system, and identified a number of cases of suspected iDILI, at a rate that is higher than previously reported from other multicenter trials. Such approaches applied across an institution or institutions could better identify and define iDILI and improve our understanding of this enigmatic condition.

## Materials and Methods

Data from subjects was collected and reviewed as part of the CMH Institutional Review Board-approved DSS ADR-detection project from January 1, 2012 to December 31, 2013. All procedures performed in studies involving human participants were in accordance with the ethical standards of the CMH Institutional Review Board and with the 1964 Helsinki declaration and its later amendments or comparable ethical standards. For this type of retrospective study, written informed consent was waived by the CMH Institutional Review Board.

As part of the ADR-detection project, an automated daily report is generated using SAP BusinessObjects (Walldorf, Germany) which searches laboratory data in the electronic medical record (EMR; Cerner, Kansas City, MO) and sorts data based on the specific criteria defined by the user. Selected criteria to detect potential DILI included serum ALT > 5x ULN and/or total serum bilirubin > 1.5x ULN which were based on clinical chemistry criteria for DILI as established by Aithal *et al*.^14^. For reference, the upper limit of normal identified at CMH for ALT is 50 U/L for all ages and for total bilirubin is 1.2 mg/dL in children older than 6 months. A dedicated clinical pharmacist then reviews the medical history of all patients meeting trigger criteria **(**Fig. 1). Patients were excluded if a known underlying diagnosis was associated with abnormal liver enzyme elevation. Identification of possible contributing medications was based on the known side effect profile for the medications the child was receiving and the drug exposure timeline relative to the triggered laboratory result. When a possible iDILI was recognized, the clinical pharmacist would contact and discuss the case with the prescribing physician. The prescribing physician determined the course of action or possible referral for further evaluation. If the drug was implicated as a possible cause of the liver injury, the ADR profile was updated to recognize the iDILI side effect including the severity assessment.

The injury relationship was further defined by application of the RUCAM^10,11^. The RUCAM assessment first defines whether an injury is “hepatocellular,” “mixed,” or “cholestatic” based on the R ratio. This ratio is calculated by dividing the ALT by the ALKP using multiples of the local upper limit of normal for both values. As such, R = (ALT value/ALT ULN)/(ALKP value/ALKP ULN). R ratios of >5 define a hepatocellular injury, <2 a cholestatic injury, and between 2 and 5 a mixed injury. The RUCAM score then categorizes patients into “definite or highly probable” (score > 8), “probable” (score 6–8), “possible” (score 3–5), “unlikely” (score 1–2) and “excluded” (score </ = 0) cases of iDILI. All cases deemed possible, probably, or definite where included in the study. Patients receiving chemotherapy and patients with acetaminophen toxicity were excluded from further evaluation after identification.
